# Supplementary material for: Endothelial-secreted Endocan activates PDGFRA and regulates vascularity and spatial phenotype in glioblastoma
Source: Nat Commun. 2025 Jan 7;16:471. doi: 10.1038/s41467-024-55487-1 (PMC11707362; doi:10.1038/s41467-024-55487-1)
Supplement: Supplementary file 1 — Supplementary Information [file 41467_2024_55487_MOESM1_ESM.pdf]

Supplementary Information

Endothelial-secreted Endocan activates PDGFR alpha and regulates vascularity, radioresistance, and regional phenotype in glioblastoma

Soniya Bastola, Marat S. Pavlyukov, Neel Sharma, Yasmin Ghochani, Mayu A. Nakano, Sree Deepthi Muthukrishnan, Sang Yul Yu, Min Soo Kim, Alireza Sohrabi, Natalia P. Biscola, Daisuke Yamashita, Ksenia S. Anufrieva, Tatyana F. Kovalenko, Grace Jung, Tomas Ganz, Beatrice O'Brien, Riki Kawaguchi, Yue Qin, Stephanie K. Seidlits, Alma L Burlingame, Juan A. Oses-Prieto, Leif A. Havton, Steven A. Goldman, Anita B. Hjelmeland, Ichiro Nakano and Harley I. Kornblum

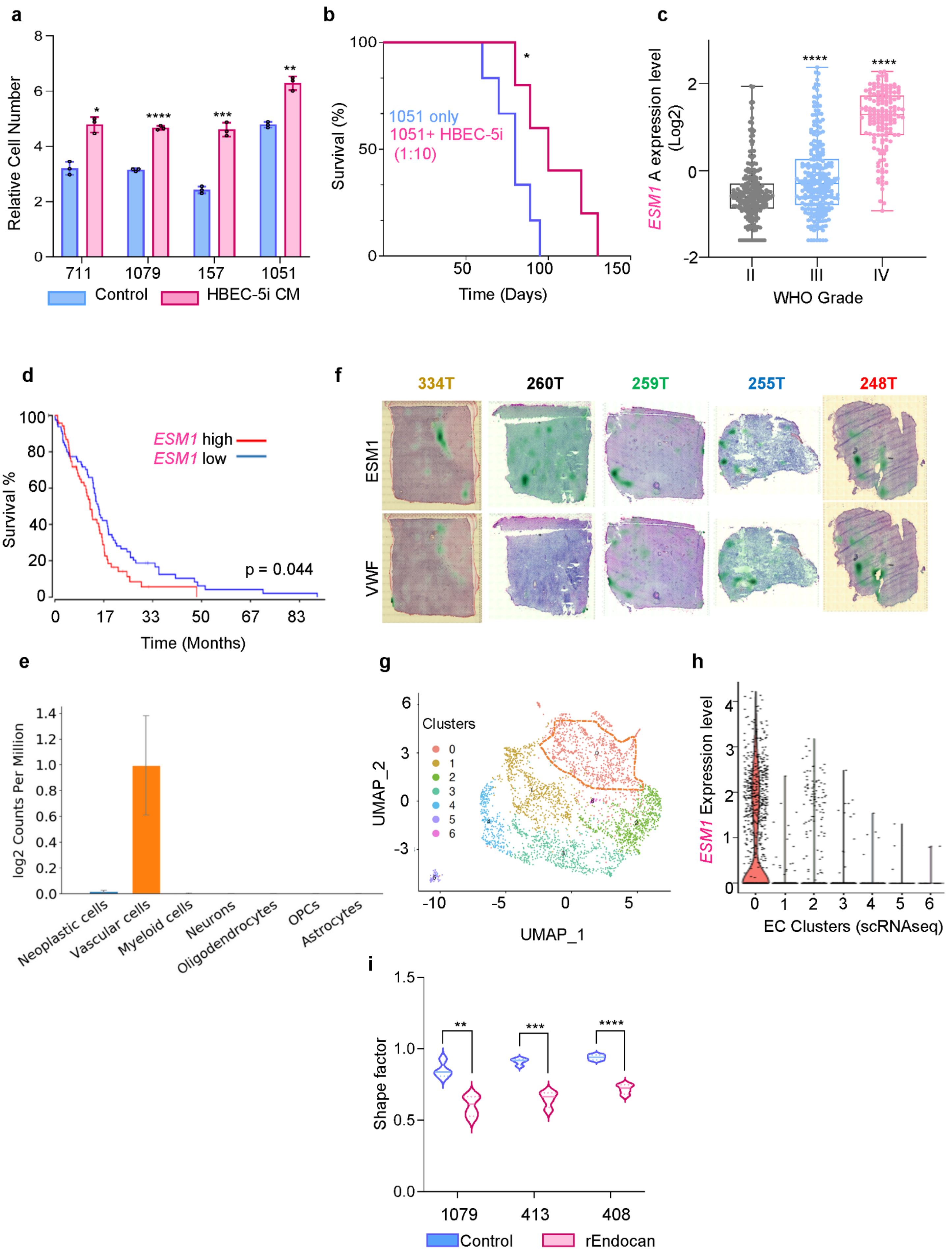

a
Relative Cell Number
711
1079
157
1051
Control
HBEC-5i CM
b
Survival (%)
1051 only
1051+ HBEC-5i
(1:10)
Time (Days)
c
ESM1 A expression level
(Log2)
WHO Grade
II
III
IV
d
Survival %
ESM1 high
ESM1 low
p = 0.044
Time (Months)
f
334T
260T
259T
255T
248T
ESM1
VWF
e
log2 Counts Per Million
Neoplastic cells
Vascular cells
Myeloid cells
Neurons
Oligodendrocytes
OPCs
Astrocytes
g
Clusters
UMAP_2
UMAP_1
h
ESM1 Expression level
EC Clusters (scRNAseq)
i
Shape factor
1079
413
408
Control
rEndocan

Supplementary Figure 1. Vascular-secreted Endocan promotes migration of GBM cells.

a, Proliferation of GBM cells (711, 1079, 157, 1051) treated with control media or conditioned media collected from Human Brain Endothelial Cells-5i (HBEC-5i) cells; n=3 biological replicates. *P=0.0017, ****P=0.0002*, *****P<0.0001*, and ***P=0.0007,* unpaired two-tailed Student's t-test.

b, Kaplan-Meier survival analysis of SCID mice intracranially injected with gliomaspheres alone (1051) or gliomaspheres with VE cells (1051+HBEC-5i) at a 10:1 ratio. (n=5 mice per group). Log-rank test, **P=0.0323*.

c, Expression of *ESM1* in TCGA's WHO grade II, III, and IV glioma tumors. One-way ANOVA followed by Tukey's post hoc test; *****P<0.0001*.

d, Kaplan-Meier analysis showing the survival of 667 glioma patients subdivided based on ESM1 expression level. Log-rank test; **P = 0.044*.

e, Analysis of the Darmanis single cell RNAseq dataset[15] showing ESM1 expression levels across different cell types within the GBM tumor, the graph was generated using the www.gbmseq.org online tool[15].

f, Expression of ESM1 and VWF in GBM samples from a previously published Visium dataset (n = 5 different patients)[16].

g, Analysis of Xie single cell RNAseq dataset[18] showing different clusters of VE cells present within the GBM tumor. Cells related to cluster 0 are highlighted.

h, Analysis of *ESM1* expression in clusters shown in "G".

i, Quantification of shape factor/circularity in rEndocan (10ng/ml) treated and control group of spheroids formed by 408, 413, or 1079 cells. Circularity was calculated using ImageJ shape description. ***P=0.0016* (1079 cells); ****P=0.0001* (413 cells); *****P<0.0001* (408 cells), unpaired two-tailed Student's t-test.

Source data are provided as a Source Data file.

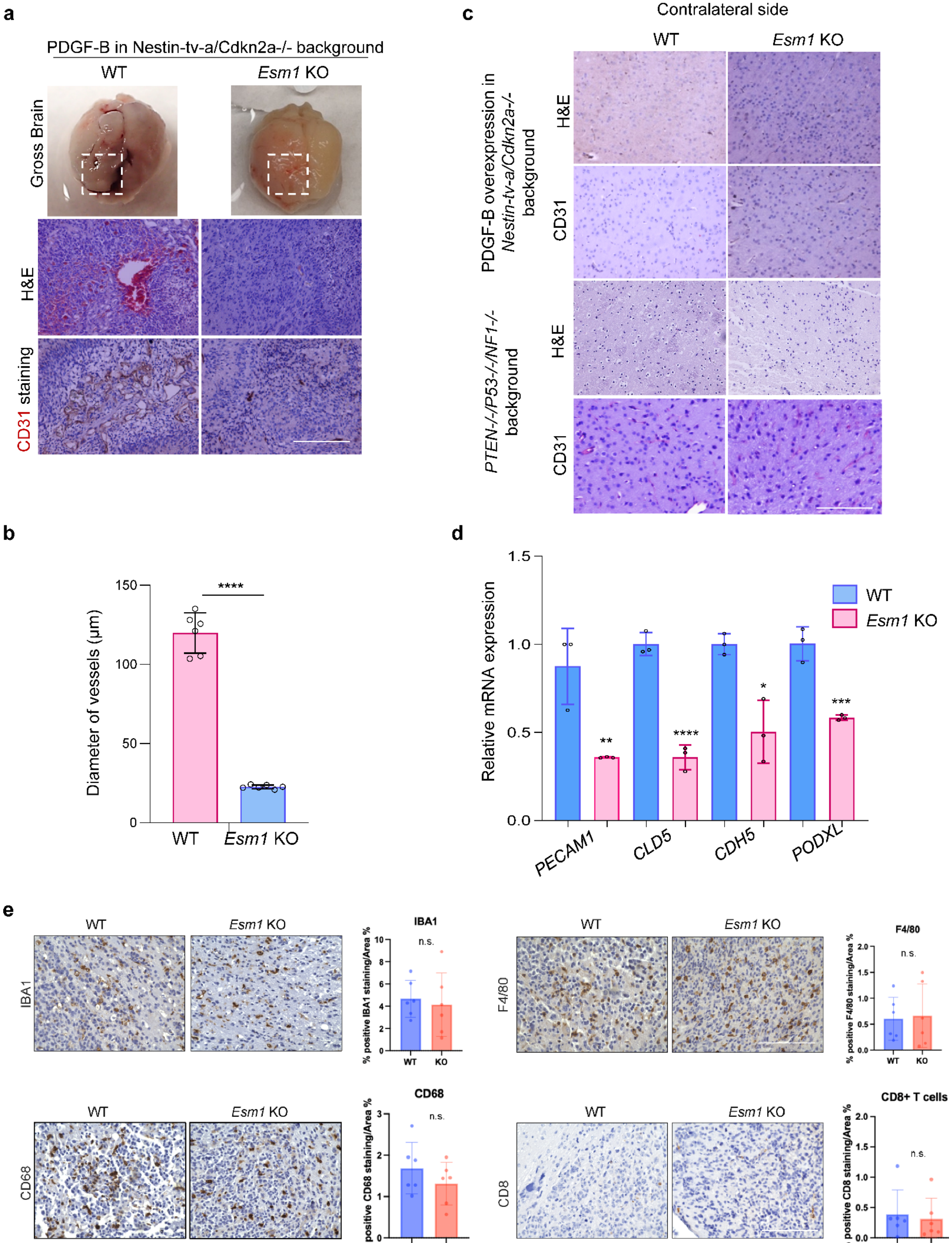
a
PDGF-B in Nestin-tv-a/Cdkn2a-/- background
WT
Esm1 KO
Gross Brain
H&E
CD31 staining
b
Diameter of vessels (μm)
****
WT
Esm1 KO
c
Contralateral side
WT
Esm1 KO
PDGF-B overexpression in Nestin-tv-a/Cdkn2a-/- background
PTEN-/-/P53-/-/NF1-/- background
H&E
CD31
d
Relative mRNA expression
WT
Esm1 KO
PECAM1
CLD5
CDH5
PODXL
e
IBA1
% positive IBA1 staining/Area %
n.s.
F4/80
% positive F4/80 staining/Area %
CD68
% positive CD68 staining/Area %
CD8+ T cells
CD8
% positive CD8 staining/Area %
KO

Supplementary Figure 2. Endocan is essential for establishing the hypervascular phenotype of GBM.

a, Tumors formed by murine glioblastoma cells (isolated from Nestin-Tva/Cdkn2a-/- mice intracranially injected with RCAS-PDGFB lentiviruses) in WT and *Esm*1 KO mice brains. Images of the brains (upper panel); H&E staining of the tumor slices (middle panel, Scale bar, 200 μm); CD31 staining (lower panel, Scale bar, 200 μm).

b, Quantitation of the diameter of the blood vessels in samples from Fig S2A as measured by CD31+ vessels, 6 regions per tumor section was measured, n=5 mice per group, *****P<0.0001,* unpaired two-tailed Student's t-test.

c, H&E staining (upper panel) and CD31 staining (lower panel) of non-tumor/contralateral brain sections obtained from tumor bearing Esm1 WT and KO mice injected with cells related to two different mouse GBM models. Scale bar, 200 μm.

d, qRT-PCR analysis of expression of vascular markers genes (Pecam1, Cld5, Cdh5, Podxl) from tumors formed in WT and Esm1 KO mice. n=3 biological replicates; **P=0.0141 (PECAM1); ****P=0.0003 (CLD5); **P=0.0101* (CDH5) and ****P=0.0017* (PODXL), unpaired two-tailed Student's t-test.

e, Microscopic images of WTD and KOD tumor sections stained for IBA1, CD68, F4/80 and CD8 using n=3 mice per group. *P=0.7016* (IBA1*), P=0.2852* (CD68), *P=0.7373* (CD8+), *P=0.8513* (F4/80), unpaired two-tailed Student's t-test.

Source data are provided as a Source Data file.

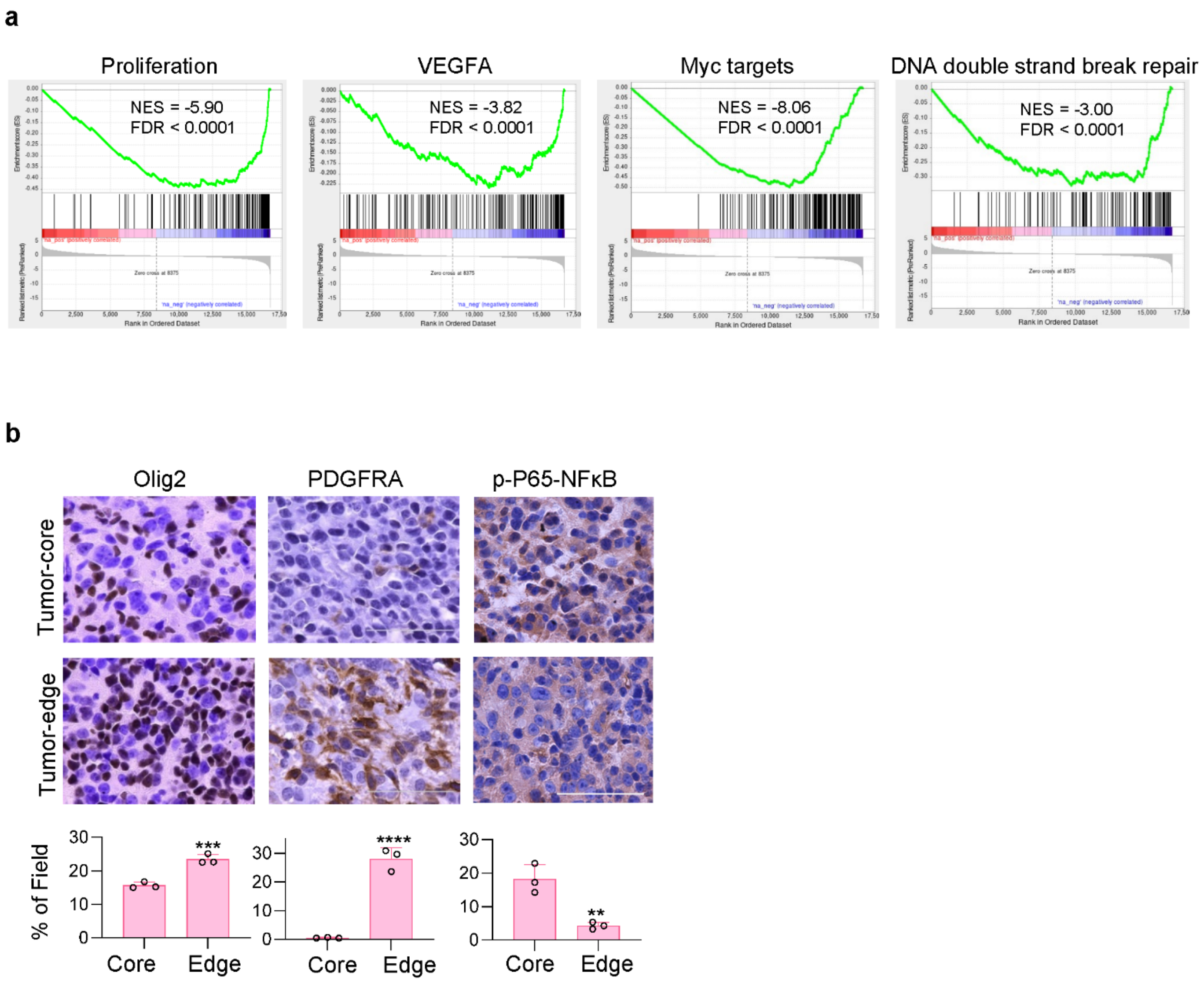

Supplementary Figure 3. Endocan regulates spatial identity of GBM cells.

a, Gene Set Enrichment Analysis (GSEA) plots comparing gene expression in tumors formed in *Esm1* KO vs. WT mice.

b, Representative IHC images of core and edge regions of the tumors obtained as in "Fig 3D" and stained for PDGFRA, Olig2 and anti-pP65-NF-κB (upper panel). % of field positive areas for the indicated proteins (lower panel). Samples from n=5 mice. Per group ****P=0.0015* (Olig2),

*****P=0. 0003* (PDGFRA), ***P=0.0062* (p-p65-NFκB), unpaired two-tailed Student's t-test. Scale bar, 200μm. Source data are provided as a Source Data file.

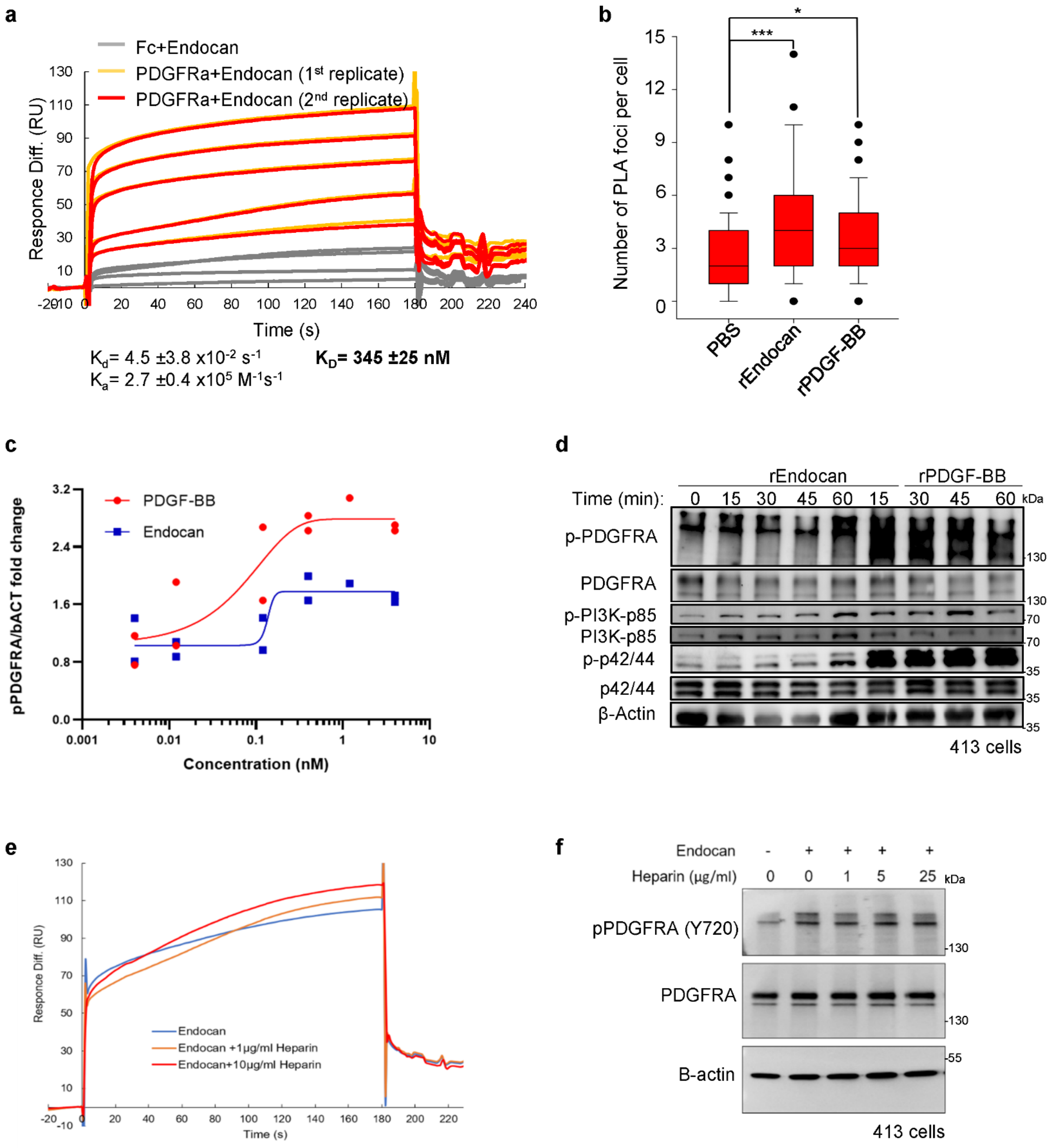
a
Fc+Endocan
PDGFRa+Endocan (1st replicate)
PDGFRa+Endocan (2nd replicate)
Responce Diff. (RU)
Time (s)
Kd= 4.5 ±3.8 x10-2 s-1
KD= 345 ±25 nM
Ka= 2.7 ±0.4 x105 M-1s-1
b
Number of PLA foci per cell
***
*
PBS
rEndocan
rPDGF-BB
c
PDGF-BB
Endocan
pPDGFRA/bACT fold change
Concentration (nM)
d
rEndocan
rPDGF-BB
Time (min): 0 15 30 45 60 15 30 45 60
kDa
p-PDGFRA
PDGFRA
p-PI3K-p85
PI3K-p85
p-p42/44
p42/44
β-Actin
413 cells
e
Endocan
Endocan +1μg/ml Heparin
Endocan+10μg/ml Heparin
Responce Diff. (RU)
Time (s)
f
Endocan - + + + +
Heparin (μg/ml) 0 0 1 5 25
kDa
pPDGFRA (Y720)
PDGFRA
B-actin
413 cells

Supplementary Figure 4. Endocan binds to and activates PDGFRA in human glioblastoma cells.

a BIAcore signal response kinetics studies of interactions between His_6-Endocan and Fc-PDGFRα (red and yellow; two biological replicates) and between His_6-Endocan and Fc (grey),

b, PDGFRA phosphorylation intensity determined by *In situ* Proximity Ligation assay (PLA) in 157 cells treated with rEndocan (10ng/ml), rPDGF-BB (10ng/ml) or PBS (negative control). **P=0.019*, ****P=0.0006,* unpaired two-tailed Student's t-test.

c, Relative level of PDGFRA phosphorylation in 1079 cells incubated with different concentrations of rEndocan or rPDGF-BB for 30 minutes. Quantification was performed using all bands shown on the Figure 4C. The experiment was performed in n = 2 biological replicates.

d, Western blot analysis of 413 cells incubated with 10ng/ml rEndocan or rPDGFBB for the indicated periods of time.

e, BIAcore signal response kinetics studies of interactions between His6-Endocan and Fc-PDGFRα in a presence or absence of the indicated concentrations of Heparin.

f, WB analysis of 413 cells incubated with rEndocan (10ng/ml) in the presence or absence of the indicated concentrations of Heparin, experiment was performed in n=3 biological replicates. Source data are provided as a Source Data file.

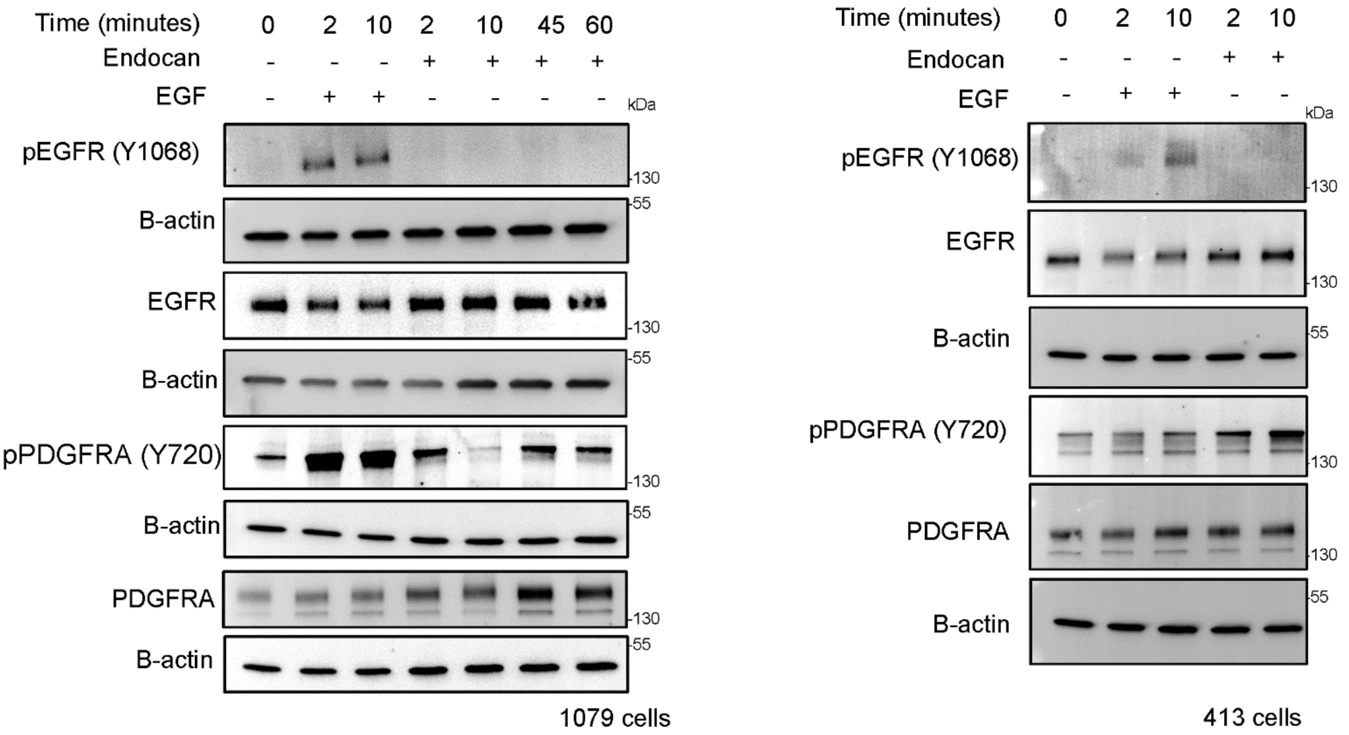

Supplementary Figure 5. Endocan activates PDGFRA but not EGFR in human glioblastoma cells.

WB analysis of 1079 (left panel) and 413 (right panel) cells incubated with rEndocan (100ng/ml) or rEGF (100ng/ml) for the indicated periods of time, representative data from n=3 biological replicates.

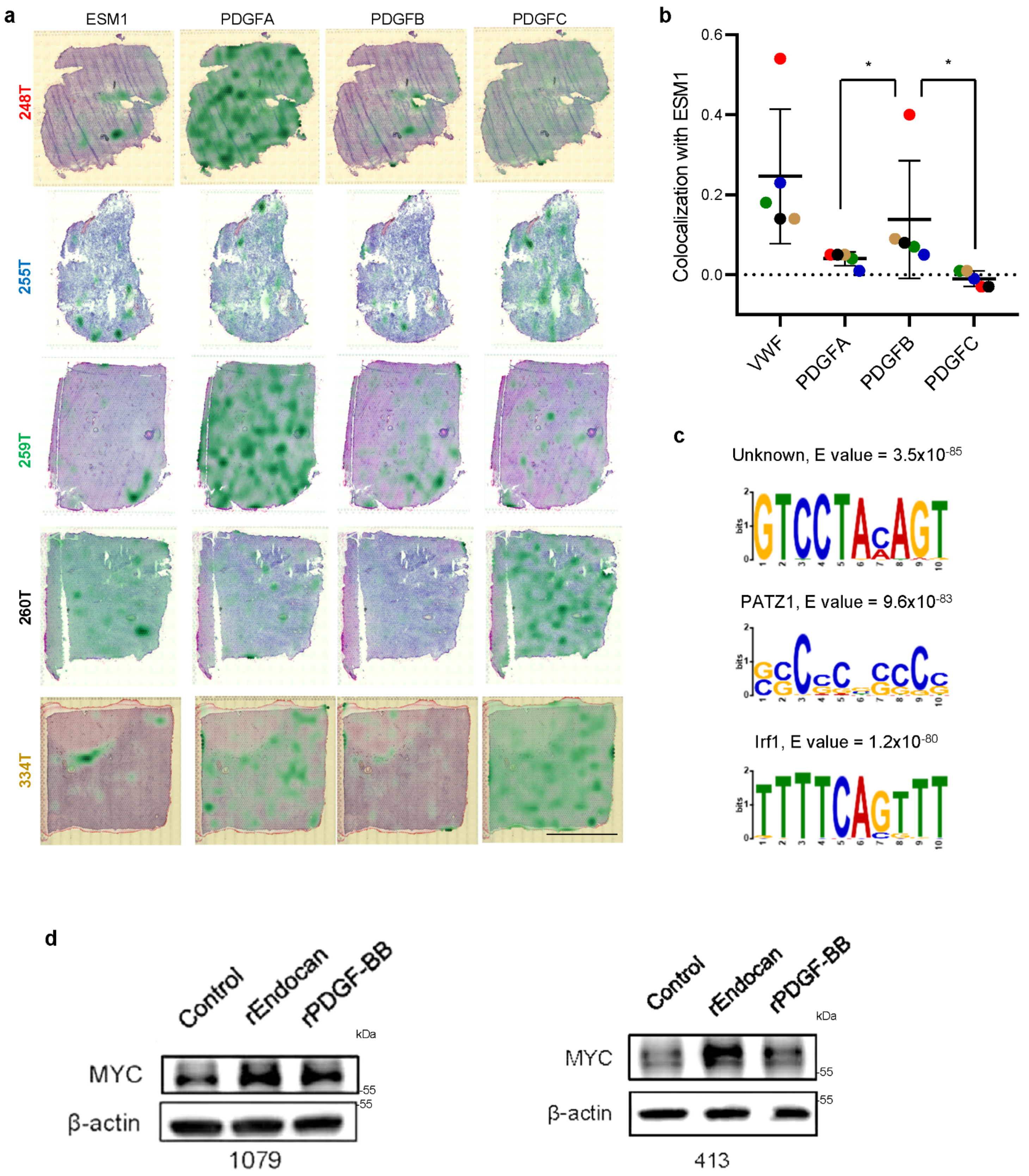
a
ESM1
PDGFA
PDGFB
PDGFC
248T
255T
259T
260T
334T
b
Colocalization with ESM1
0.6
0.4
0.2
0.0
*
*
VWF
PDGFA
PDGFB
PDGFC
c
Unknown, E value = 3.5x10-85
PATZ1, E value = 9.6x10-83
Irf1, E value = 1.2x10-80
d
Control
rEndocan
rPDGF-BB
kDa
MYC
β-actin
55
55
1079
Control
rEndocan
rPDGF-BB
kDa
MYC
β-actin
55
55
413

Supplementary Figure 6. Endocan upregulates *Myc* expression.

a, Expression of ESM1, VWF, PDGFA, PDGFB and PDGFC in GBM samples from previously published Visium dataset (Neurooncol Adv. 2023, 5(1):vdad142) (n = 5 different patients).

b, Quantification of ESM1 expression colocalization with the expression of VWF, PDGFA, PDGFB and PDGFC in samples as in “B”. Data points related to different patients are indicated in different colors. Wilcoxon signed rank-sum test, *$P<0.05$.

c, DNA motifs over-represented in the ATACseq dataset generated from tumors derived from *Esm1* WT and KO mice (n=2 mice per group). Transcription factors that bind to the corresponding motif and E values are indicated.

d, Western blot analysis of 1079 and 413 cells treated with 10ng/ml rEndocan and rPDGF-BB for 72 hours. n=3 biological replicates.

Source data are provided as a Source Data file.

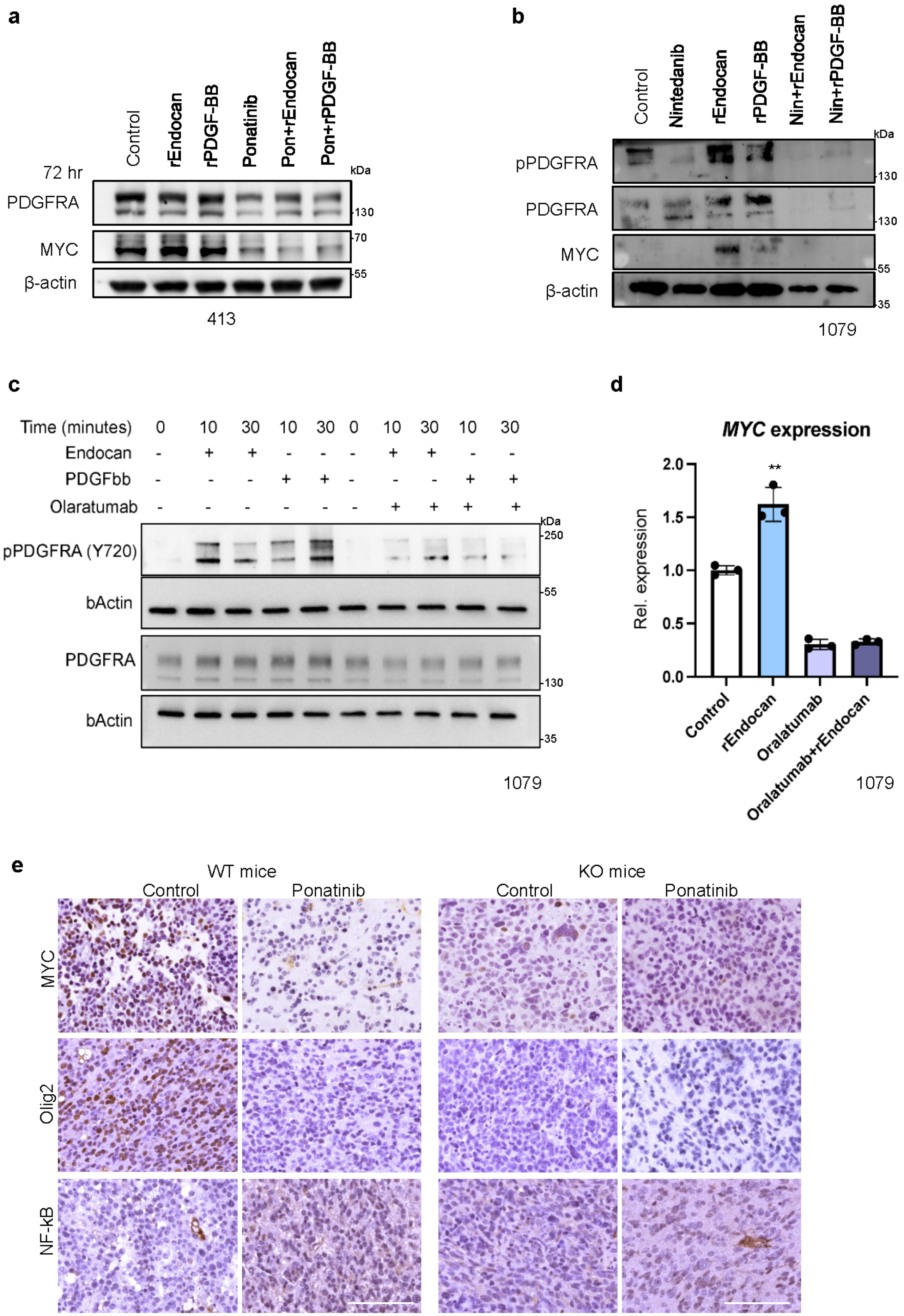
a
Control
rEndocan
rPDGF-BB
Ponatinib
Pon+rEndocan
Pon+rPDGF-BB
72 hr
kDa
PDGFRA
130
MYC
70
β-actin
55
413
b
Control
Nintedanib
rEndocan
rPDGF-BB
Nin+rEndocan
Nin+rPDGF-BB
kDa
pPDGFRA
130
PDGFRA
130
MYC
55
β-actin
35
1079
c
Time (minutes) 0 10 30 10 30 0 10 30 10 30
Endocan - + + - - - + + - -
PDGFbb - - - + + - - - + +
Olaratumab - - - - - - + + + +
kDa
pPDGFRA (Y720)
250
bActin
55
PDGFRA
130
bActin
35
1079
d
MYC expression
**
Rel. expression
2.0
1.5
1.0
0.5
0.0
Control
rEndocan
Oralatumab
Oralatumab+rEndocan
1079
e
WT mice
KO mice
Control
Ponatinib
Control
Ponatinib
MYC
Olig2
NF-kB

Supplementary Figure 7. Inhibition of PDGFRA pathway attenuates effect of Endocan on GBM cells.

a, Western blot analysis of 413 cells treated with 10ng/ml of rEndocan, rPDGF-BB, ponatinib (1μM) (Pon) and their combination treatment for 72 hours, Representative result of n=3 biological replicates.

b, Western blot analysis of 1079 cells treated with rEndocan (10ng/ml), rPDGF-BB (10ng/ml), Nintedanib (1μM) and their combination treatment for 72 hours. Representative result of n=3 biological replicates.

c, Western blot analysis of 1079 cells incubated with 10ng/ml of rEndocan or 5ng/mL of rPDGFbb for the indicated period of time in the presence or absence of olaratumab (9 nM). Representative result of n=3 biological replicates.

d, qRT-PCR analysis of MYC expression in 1079 cells cultivated for 3 days with rEndocan(10ng/ml) in the presence or absence of olaratumab (9 nM). n=3 independent experiments **P=0.0029, rEndocan vs. Control, unpaired two-tailed Student's t-test.

e, Representative IHC staining for MYC, Olig2, and P65-NF-κB of tumors formed by 7080 cells in *Esm1* WT or KO mice that were treated with ponatinib or the solvent as control. Scale bar, 100μm. n=5 mice per group.

Source data are provided as a Source Data file.

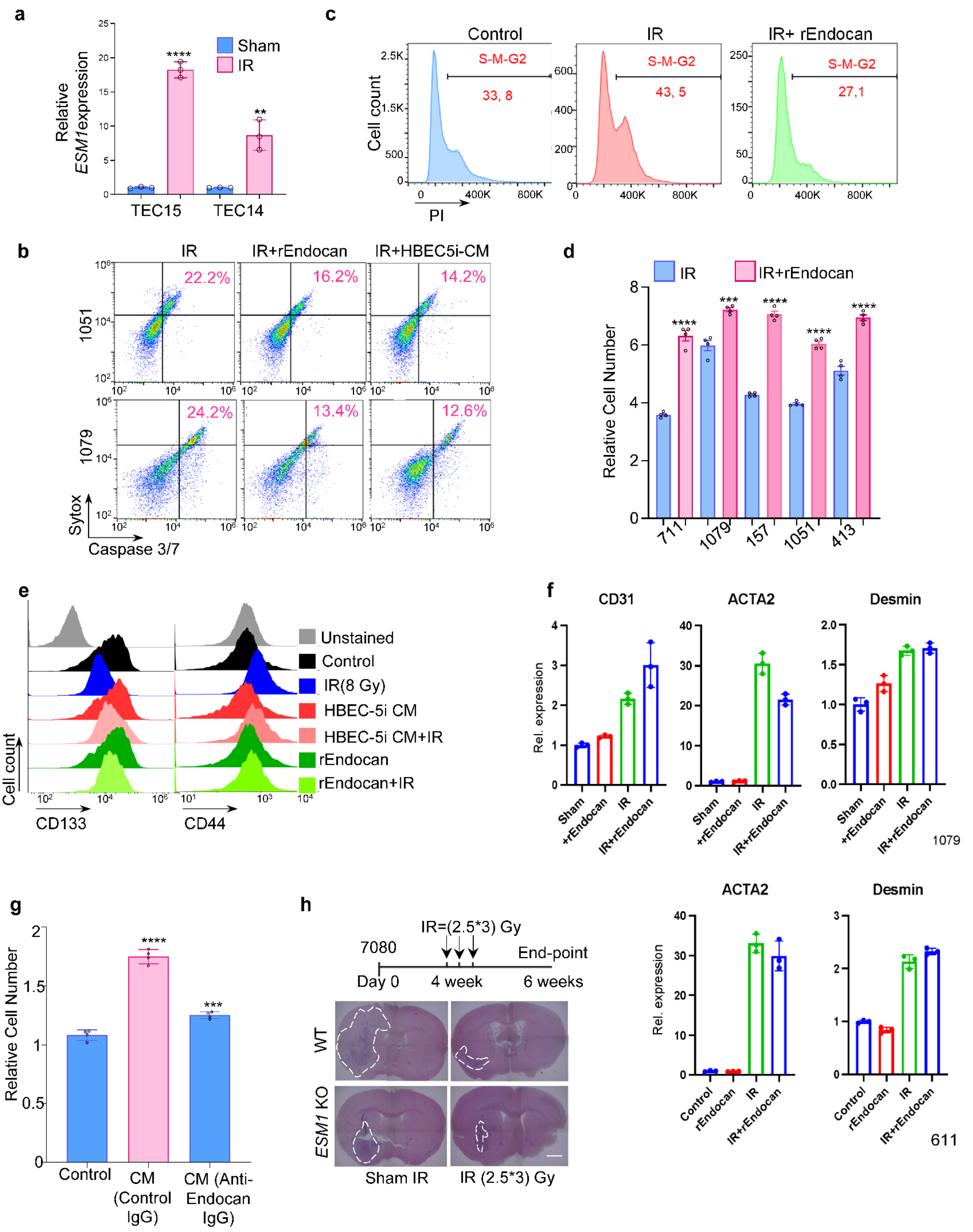
a
Relative ESM1expression
Sham
IR
****
**
TEC15
TEC14
c
Control
IR
IR+ rEndocan
S-M-G2
33, 8
43, 5
27,1
Cell count
PI
b
IR
IR+rEndocan
IR+HBEC5i-CM
22.2%
16.2%
14.2%
24.2%
13.4%
12.6%
1051
1079
Sytox
Caspase 3/7
d
IR
IR+rEndocan
Relative Cell Number
711
1079
157
1051
413
e
Unstained
Control
IR(8 Gy)
HBEC-5i CM
HBEC-5i CM+IR
rEndocan
rEndocan+IR
Cell count
CD133
CD44
f
CD31
ACTA2
Desmin
Rel. expression
Sham
+rEndocan
IR
IR+rEndocan
1079
g
Relative Cell Number
Control
CM (Control IgG)
CM (Anti-Endocan IgG)
h
IR=(2.5*3) Gy
7080
End-point
Day 0
4 week
6 weeks
WT
ESM1 KO
Sham IR
IR (2.5*3) Gy
ACTA2
Desmin
Rel. expression
Control
rEndocan
IR
IR+rEndocan
611

Supplementary Figure 8. Endocan protects glioblastoma cells from radiotherapy.

A, qRT-PCR analysis of *ESM1* gene expression in TEC15 and TEC14 VE cells 48 hours after treatment with 8 Gy radiation; n=3 biological replicates, ****$P<0.0001$ (TEC15) and *P=0.0039 (TEC14), unpaired two-tailed Student's t-test.

B, FACS analysis of caspase 3/7 and SYTOX staining of 1051 cells that were preincubated with rEndocan or HBEC-5i CM for 3 days, irradiated with 8 Gy and stained two days later.

C, FACS analysis of cell cycle distribution of 1051 cells that were left untreated, irradiated with 8Gy or preincubated for 3 days with rEndocan (10ng/ml) and subsequently irradiated.

D, *In vitro* radio-sensitivity assay of gliomaspheres (711, 1079, 157, 1051, and 413) pretreated with rEndocan (10ng/ml) for 3 days and irradiated with 8Gy. Cell growth was measured on day 5 after irradiation. n=4 biological replicates, ****$P<0.0001$ (711), ***$P=0.0006$ (1079), ****$P<0.0001$ (157), ****$P<0.0001$ (1051), and ****$P<0.0001$ (413), unpaired two-tailed Student's t-test.

E, FACS analysis of CD133 and CD44 staining of 1079 cells pretreated with rEndocan (10ng/ml) or HBEC-5i CM for 3 days, irradiating with 8Gy and stained two days later. Unstained cells were used as control. ***$P<0.001$, ****$P<0.0001$, unpaired two-tailed Student's t-test.

F, qRT-PCR analysis of CD31, ACTA2 and Desmin expression in non-irradiated or irradiated (8Gy) gliomaspheres (1079 and 611) cultivated in the presence or absence of rEndocan (10ng/ml). n=3 biological replicates.

G, *In vitro* radio-sensitivity assay of 1079 cells preincubated for 3 days with control media, or HBEC-5i CM that was incubated with Endocan blocking antibody (Endocan depleted CM), or isotype control antibody. After incubation cells were irradiated at 8 Gy and analyzed after 3 more days. n=4 biological replicates. ****$P<0.0001$ (Control IgG). ***$P=0.0008$ (Endocan depleted CM), unpaired two-tailed Student's t-test.

H, Representative H&E images of brain sections obtained from *Esm1* WT or *Esm1* KO mice intracranially injected with 7080 cells and subsequently irradiated with 3 doses of 2.5 Gy 14 days after injection. n=5 mice per group. Scale bar, 500µm.

Source data are provided as a Source Data file.

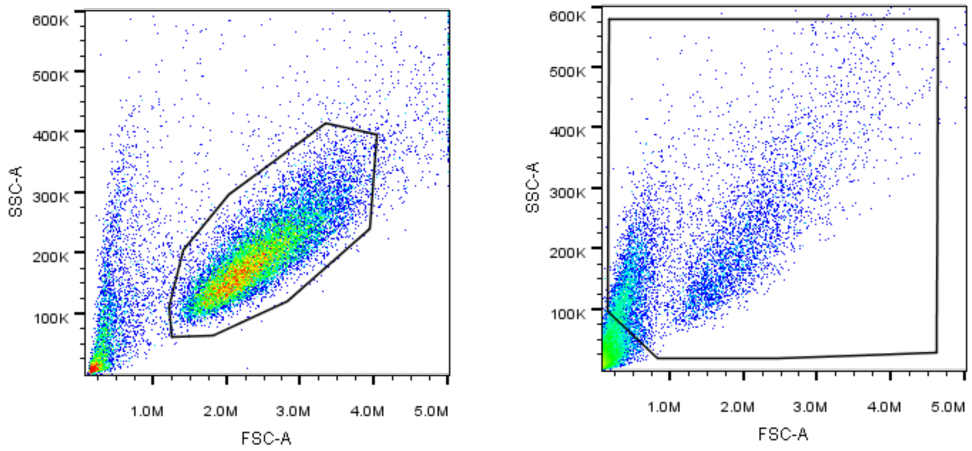

Supplementary Figure 9. Flow cytometry gating strategies.

FACS gating strategy used for all assays except apoptosis analysis (left panel) and gating strategy was used for apoptosis analysis (right panel).

Uncropped scans of blots from Supplementary Figures

Sup fig 4c

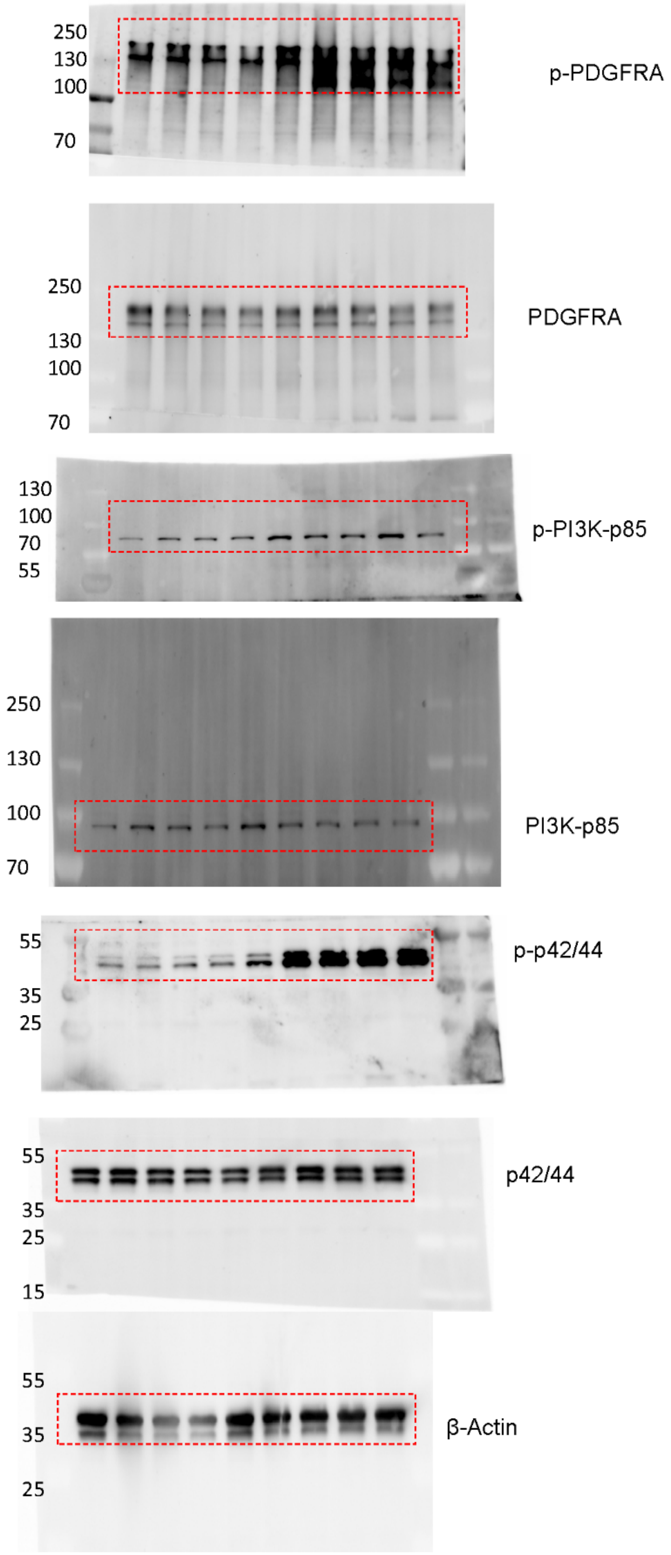

Sup fig 4f

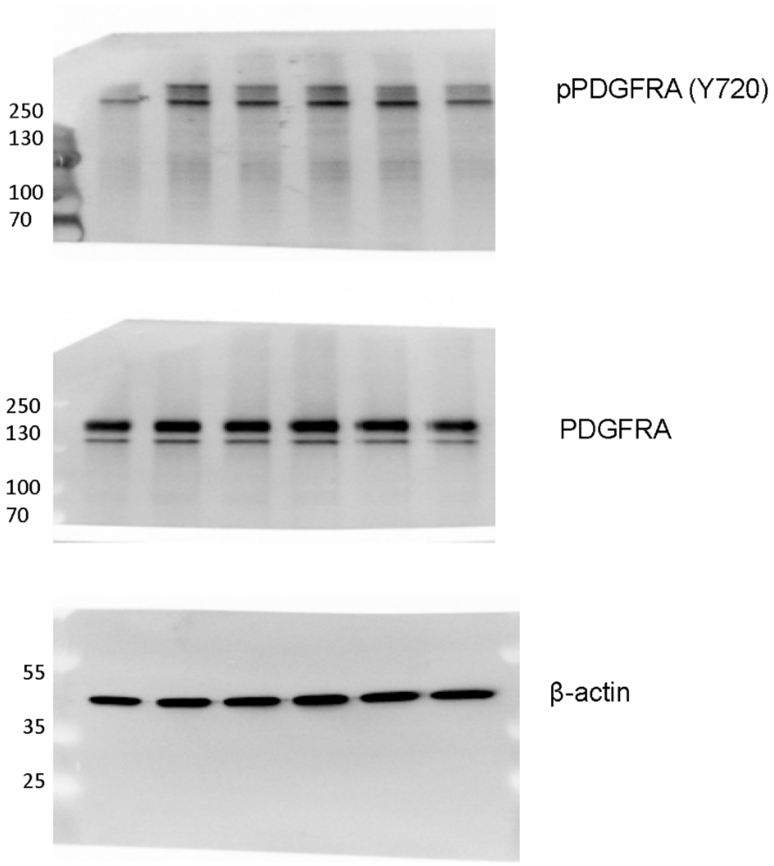
250
130
100
70
pPDGFRA (Y720)
250
130
100
70
PDGFRA
55
35
25
β-actin

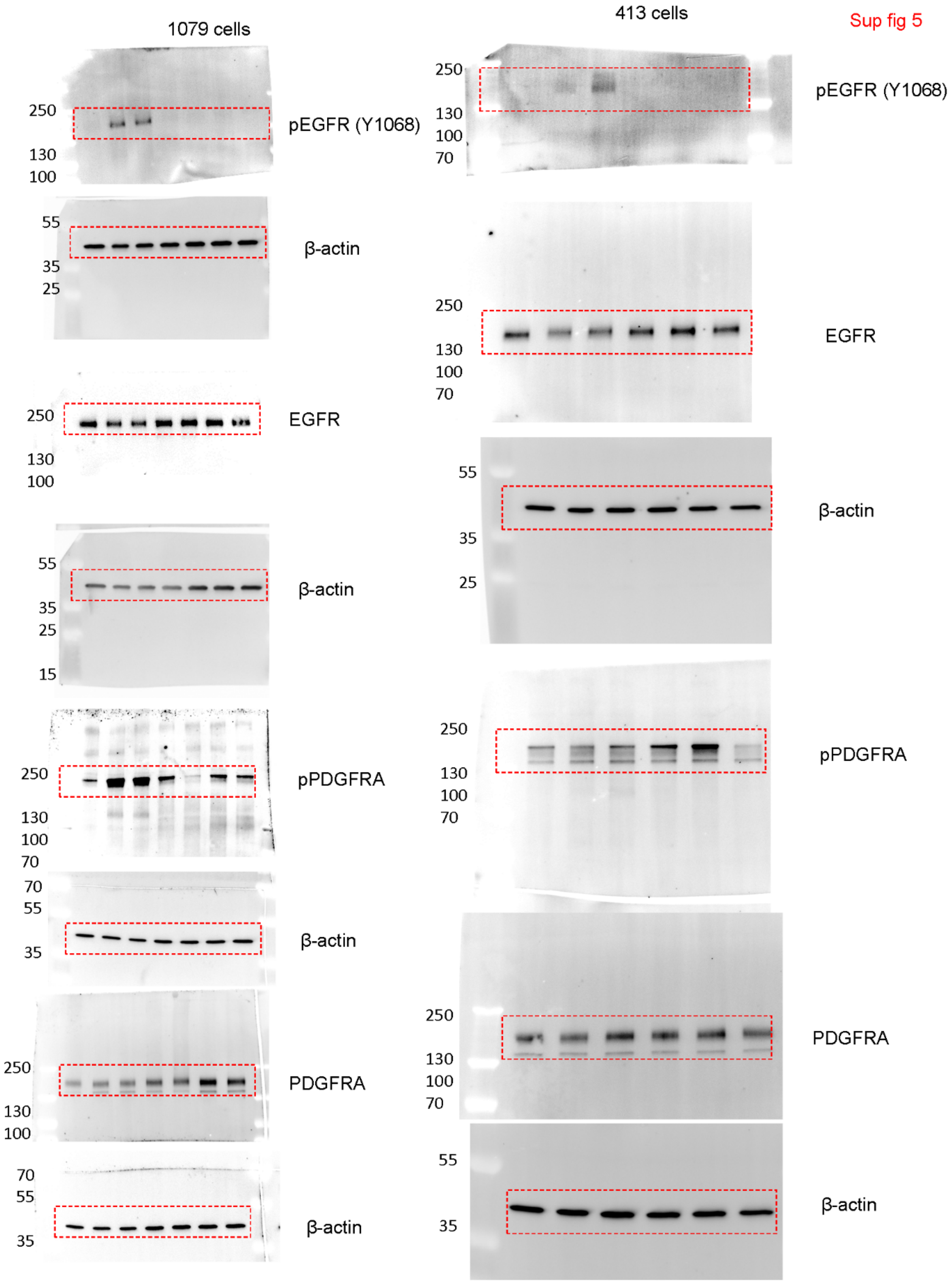
1079 cells
413 cells
250
130
100
pEGFR (Y1068)
55
35
25
β-actin
EGFR
15
pPDGFRA
70
PDGFRA

Sup fig 5

Sup fig 6d

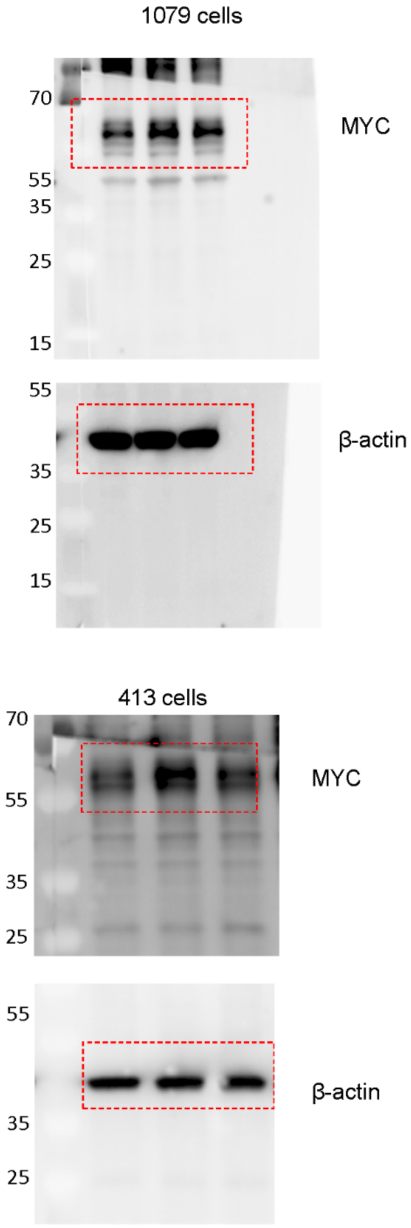
1079 cells
70
55
35
25
15
MYC
55
35
25
15
β-actin
413 cells
70
55
35
25
MYC
55
35
25
β-actin

Sup Fig 7a

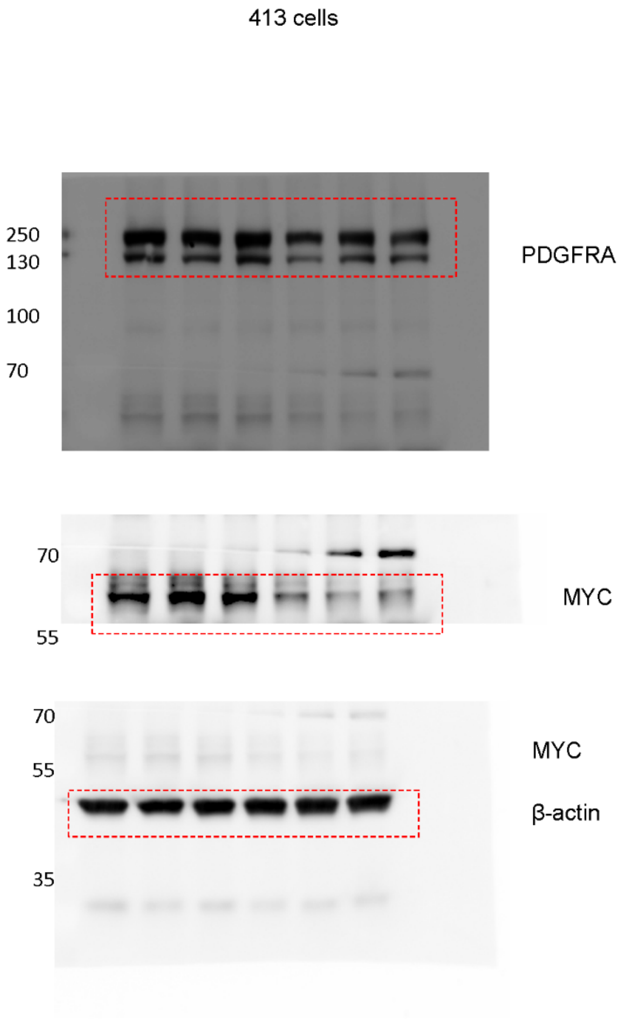

Sup Fig 7b

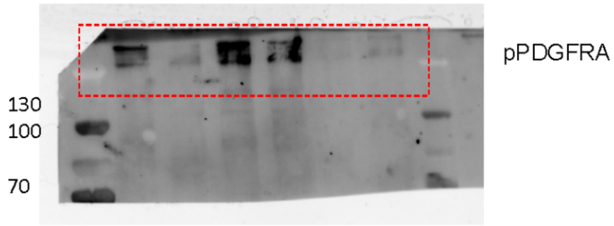
pPDGFRA
130
100
70

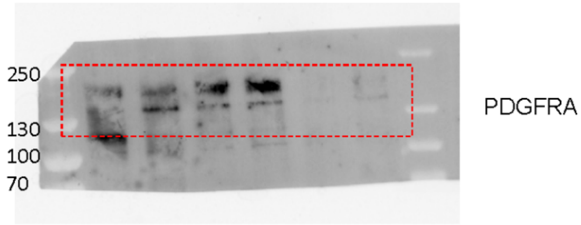
PDGFRA
250
130
100
70

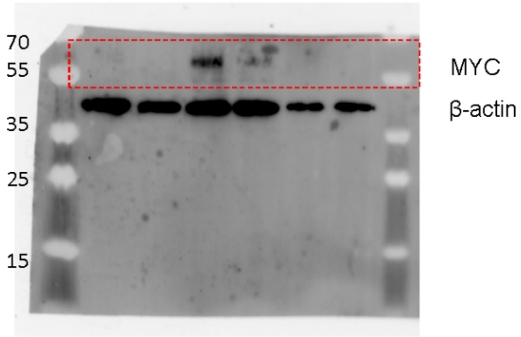
MYC
β-actin
70
55
35
25
15

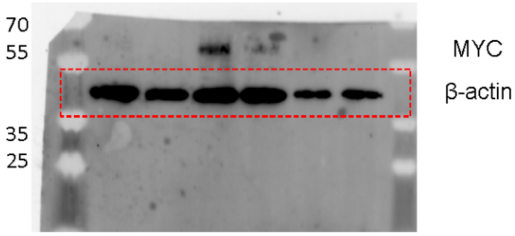
MYC
β-actin
70
55
35
25

Sup Fig 7c

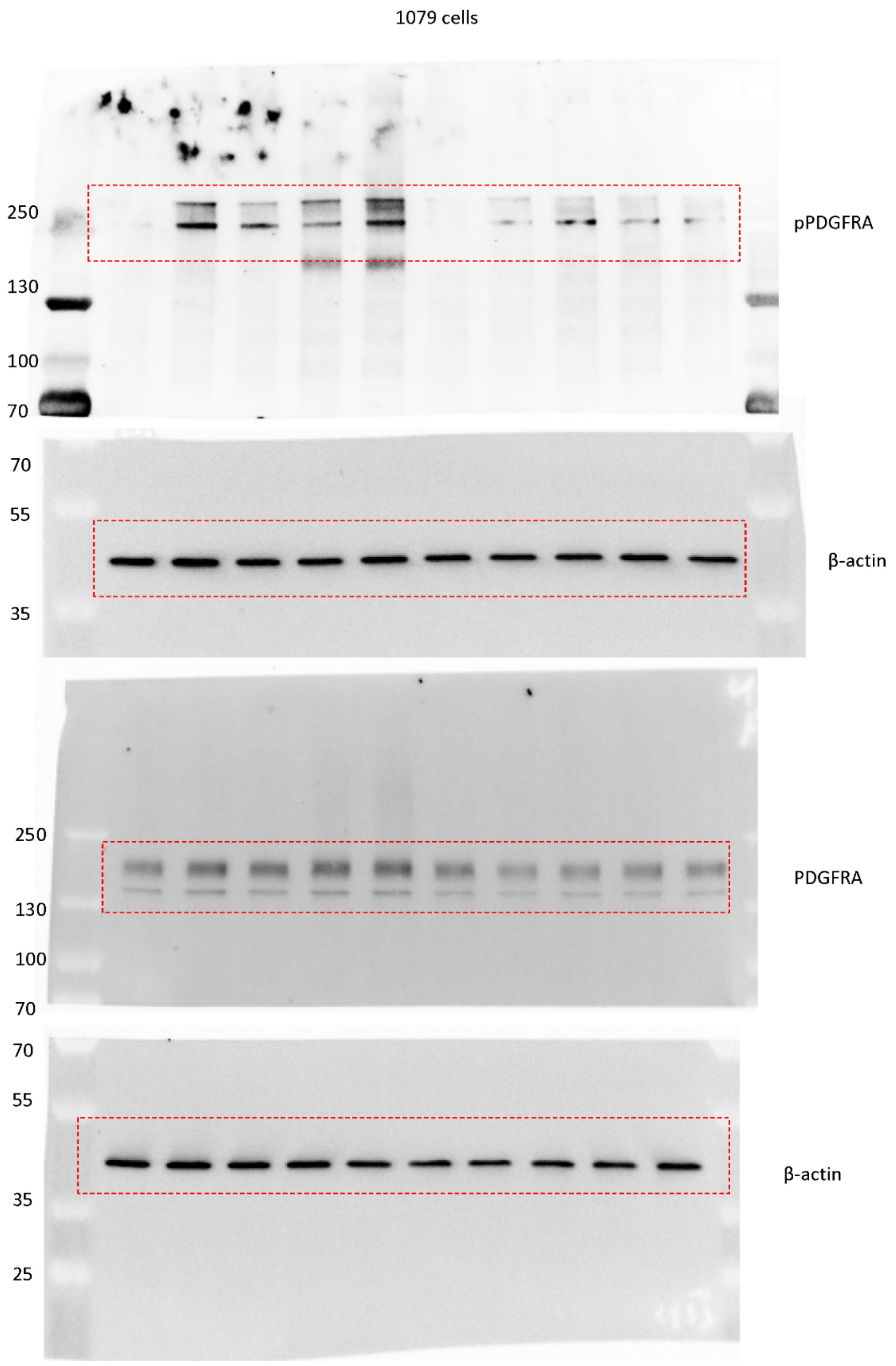
1079 cells
250
130
100
70
pPDGFRA
70
55
35
β-actin
250
130
100
70
PDGFRA
70
55
35
25
β-actin
