# Supplementary material for: Endothelial-secreted Endocan activates PDGFRA and regulates vascularity and spatial phenotype in glioblastoma
Source: Nat Commun. 2025 Jan 7;16:471. doi: 10.1038/s41467-024-55487-1 (PMC11707362; doi:10.1038/s41467-024-55487-1)
Supplement: Supplementary file 2 — Description of Additional Supplementary Files [file 41467_2024_55487_MOESM2_ESM.pdf]

Description of Additional Supplementary Files

Supplementary Data 1 (Related to Figure 2). RNA sequencing results of tumors formed by 7080 cells in ESM1 WT and KO mice

Supplementary Data 2 (Related to Figure 4). Results of LC-MS/MS identification of the proteins that were bound to recombinant His-Endocan or a control beads.

Supplementary Data 3 (Related to Figure 5). ATAC sequencing results of tumors formed by 7080 cell in Esm1 WT and Esm1 KO mice.

Supplementary Data 4 (Related to Figure 7). RNA sequencing results of human GBM cells that were preincubated with rEndocan or CM from HBEC5i cells and subsequently irradiated with 8Gy or left untreated.

Supplementary Data 5 (Related to Methods). Characterization of glioma cell lines used in this study.

Supplementary Data 6 (Related to Methods). List of oligonucleotides and their sequences used in this study.

Supplementary Data 7 (Related to Methods). List of antibodies used in this study.
