# Supplementary material for: Endothelial-secreted Endocan activates PDGFRA and regulates vascularity and spatial phenotype in glioblastoma
Source: Nat Commun. 2025 Jan 7;16:471. doi: 10.1038/s41467-024-55487-1 (PMC11707362; doi:10.1038/s41467-024-55487-1)
Supplement: Supplementary file 4 — Reporting Summary [file 41467_2024_55487_MOESM4_ESM.pdf]

nature portfolio

Corresponding author(s): Harley I. Kornblum

Last updated by author(s): 12/4/2024

Reporting Summary

Nature Portfolio wishes to improve the reproducibility of the work that we publish. This form provides structure for consistency and transparency in reporting. For further information on Nature Portfolio policies, see our Editorial Policies and the Editorial Policy Checklist.

Statistics

For all statistical analyses, confirm that the following items are present in the figure legend, table legend, main text, or Methods section.

n/a	Confirmed	
☐	☒	The exact sample size (*n*) for each experimental group/condition, given as a discrete number and unit of measurement
☐	☒	A statement on whether measurements were taken from distinct samples or whether the same sample was measured repeatedly
☐	☒	The statistical test(s) used AND whether they are one- or two-sided<br>*Only common tests should be described solely by name; describe more complex techniques in the Methods section.*
☒	☐	A description of all covariates tested
☒	☐	A description of any assumptions or corrections, such as tests of normality and adjustment for multiple comparisons
☐	☒	A full description of the statistical parameters including central tendency (e.g. means) or other basic estimates (e.g. regression coefficient) AND variation (e.g. standard deviation) or associated estimates of uncertainty (e.g. confidence intervals)
☐	☒	For null hypothesis testing, the test statistic (e.g. *F*, *t*, *r*) with confidence intervals, effect sizes, degrees of freedom and *P* value noted<br>*Give P values as exact values whenever suitable.*
☒	☐	For Bayesian analysis, information on the choice of priors and Markov chain Monte Carlo settings
☒	☐	For hierarchical and complex designs, identification of the appropriate level for tests and full reporting of outcomes
☐	☒	Estimates of effect sizes (e.g. Cohen's *d*, Pearson's *r*), indicating how they were calculated

Our web collection on statistics for biologists contains articles on many of the points above.

Software and code

Policy information about availability of computer code

Data collection	Immunofluorescence images were obtained using LEICA LASX microscope application Suite X and EVOS FL Auto Imaging system.
Data analysis	Data analysis was done using standard protocols and parameters found in the Methods section of the manuscript.<br>Flow cytometry analysis: FlowJo 10;Image analysis: FIJI2.3.0, statistics: GraphPad Prism V10, Bulk RNA-seq: STAR alignment, software DESeq, Edge R ; For GSEA analysis, mSigDB ver7.0 was used.<br>ATACseq: Picard Tools, Homer 4.11.1, Diffbind 2.16.2 was used.

For manuscripts utilizing custom algorithms or software that are central to the research but not yet described in published literature, software must be made available to editors and reviewers. We strongly encourage code deposition in a community repository (e.g. GitHub). See the Nature Portfolio guidelines for submitting code & software for further information.

Data

Policy information about availability of data

All manuscripts must include a data availability statement. This statement should provide the following information, where applicable:

- Accession codes, unique identifiers, or web links for publicly available datasets
- A description of any restrictions on data availability
- For clinical datasets or third party data, please ensure that the statement adheres to our policy

ATACseq data of the tumors formed by 7080 cells in ESM1 WT and KO mice were deposited to the Gene Expression Omnibus (GEO) under the accession number

GSE137796 [https://www.ncbi.nlm.nih.gov/geo/query/acc.cgi?acc=GSE137796]. Data for figure 1C is available to access from Ghochani et al, 2022, DOI: 10.1016/j.celrep.2022.111511 [https://www.ncbi.nlm.nih.gov/geo/query/acc.cgi?acc=GSE186932]. RNAseq data of the tumors formed by 7080 cells in ESM1 WT and KO mice were deposited to GEO under the accession number GSE137808 [https://www.ncbi.nlm.nih.gov/geo/query/acc.cgi?acc=GSE137808]. RNAseq data of patient-derived glioma sphere lines (1051 and 1079) are deposited with access codes GSE277011 [https://www.ncbi.nlm.nih.gov/geo/query/acc.cgi?acc=GSE277011] and GSE277014 [https://www.ncbi.nlm.nih.gov/geo/query/acc.cgi?acc=GSE277014]. Raw Mass spectrometry data have been deposited in ProteomeXchange via the PRIDE with the primary accession code PXD057635 https://proteomecentral.proteomexchange.org/ui?search=PXD057635. Visium data from https://pubmed.ncbi.nlm.nih.gov/38077210/ was analyzed for Figure s1F. Single-cell data from https://www.sciencedirect.com/science/article/pii/S2211124717314626 was used for s1E.
Source data are provided with this paper. Single-cell data for Figure s1G, H was used from https://insight.jci.org/articles/view/150861.
Please contact correseponding author (hkornblum@mednet.ucla.edu) if there are requests.

Research involving human participants, their data, or biological material

Policy information about studies with human participants or human data. See also policy information about sex, gender (identity/presentation), and sexual orientation and race, ethnicity and racism.

Reporting on sex and gender	Due to deidentified nature of the content of the tumor samples and cell lines used in the study, no sex and gender-based analyses were performed.
Reporting on race, ethnicity, or other socially relevant groupings	N/A
Population characteristics	N/A
Recruitment	N/A
Ethics oversight	Patient tissue samples used in this study was de-identified and collected under informed consent and approved by UCLA and UAB Medical Institutional Review Board.

Note that full information on the approval of the study protocol must also be provided in the manuscript.

Field-specific reporting

Please select the one below that is the best fit for your research. If you are not sure, read the appropriate sections before making your selection.

- [x] Life sciences
- [ ] Behavioural & social sciences
- [ ] Ecological, evolutionary & environmental sciences

For a reference copy of the document with all sections, see nature.com/documents/nr-reporting-summary-flat.pdf

Life sciences study design

All studies must disclose on these points even when the disclosure is negative.

Sample size	Sample size was determined based on standards in the field and by the effect size between groups.
Data exclusions	No data was excluded from the analyses.
Replication	Replication was performed for all experiments with a minimum of 2-3 independent times with successful replication of the data.
Randomization	For in vivo study, female mice with tumors were randomized for drug or radiation treatments. For in vitro studies, samples were randomly allocated in experimental group.
Blinding	Experiments requiring cell counting on immunostained tumor sections between control and treatment groups were blinded. For ATAC sequencing experiments, Bioinformatician performed processing anf alanysis in blinded fashion.For in vitro experiments, no adjustment was performed for all conditons.

Reporting for specific materials, systems and methods

We require information from authors about some types of materials, experimental systems and methods used in many studies. Here, indicate whether each material, system or method listed is relevant to your study. If you are not sure if a list item applies to your research, read the appropriate section before selecting a response.

Materials & experimental systems

n/a	Involved in the study
☐	☒ Antibodies
☐	☒ Eukaryotic cell lines
☒	☐ Palaeontology and archaeology
☐	☒ Animals and other organisms
☒	☐ Clinical data
☒	☐ Dual use research of concern
☒	☐ Plants

Methods

n/a	Involved in the study
☒	☐ ChIP-seq
☐	☒ Flow cytometry
☒	☐ MRI-based neuroimaging

Antibodies

Antibodies used	Anti-ALDH1A3 Sigma-Aldrich Cat#SAB1300933, RRID:AB_10607145<br>Anti-phospho AKT (Ser473) Cell Signaling Technology Cat# 9271, RRID:AB_329825<br>Anti-AKT Cell Signaling Technology Cat# 9272, RRID:AB_329827<br>Anti-β-ACTIN Cell Signaling Technology Cat# 2118; RRID:AB_561053<br>Anti-CD133-APC Miltenyi Biotec Cat#130-090-826;RRID: AB_244340<br>Anti-CD31 Abcam Cat# ab28364, RRID: AB_726362<br>Anti-CD31 Novus Cat#NB600-562, RRID:AB_10002476<br>Anti-CD44 Cell Signaling Technology Cat# 3578; RRID: AB_2076463<br>Anti-CD44-FITC BioLegend Cat# 338804, RRID:AB_1501197<br>Endocan blocking antibody R&D systems Cat# AF1810-SP<br>Anti-ESM1 Abcam Cat# ab56914, RRID:AB_941479<br>Anti-ESM1 Bioss Cat#bs-3615R, RRID:AB_10857499<br>GAPDH Abcam Cat# ab9483; RRID: AB_307273<br>Anti-Goat IgG control Thermofisher Scientific Catalog # 02-6202<br>Anti-GFP Abcam Cat# ab290; RRID:AB_303395<br>Anti-phospho GSK3β (Thr 390) Bioss Cat#bs-3148R, RRID:AB_10857056<br>Anti-HIF1α Novus Cat#NB100-105, RRID:AB_10001154<br>Anti-His-tag Cell Signaling Technology Cat# 12698, RRID:AB_2744546<br>Rabbit IgG Isotype Control Thermofisher Scientific Cat# 02-6102, RRID:AB_2532938<br>Anti-phospho-p44/42 MAPK Cell Signaling Technology Cat# 9101, RRID:AB_331646<br>Anti-p44/42 MAPK Cell Signaling Technology Cat# 9102, RRID:AB_330744<br>Anti-mCHERRY Novus Cat#NBP2-25157, RRID:AB_2753204<br>Anti-MYC Santa Cruz Biotechnology Cat# SC-40; RRID: AB_627268<br>Anti-MYC Cell Signaling Technology Cat# 13987<br>Anti-p65-NF-κB(pS536) Abcam Cat# ab86299, RRID:AB_1925243<br>Anti-OLIG2 Millipore Cat# AB9610; RRID: AB_570666<br>Anti-PI3K p85 / p55, phospho (Tyr199) Bioworld Technology Cat# BS4605, RRID: AB_1663852<br>Anti-PI3K Cell Signaling Technology Cat# 4292, RRID:AB_329869<br>Anti-phospho PDGFRα (y720) Abcam Cat#ab134068;RRID:AB_2921264<br>Anti-PDGFRα Cell Signaling Technology Cat# 3174, RRID:AB_2162345<br>Anti-phospho-Histone H2A.X (Ser139) Cell Signaling Technology Cat# 2577, AB_2118010<br>Anti-PDGFRα (C-terminal) Sigma-Aldrich Cat#SAB1404186, RRID:AB_10737660<br>Anti-YKL-40/CHI3L1 Abcam Cat#:Ab77528; RRID:AB_2040911<br>Anti-EGFR antibody CST Cat#4267<br>Anti-Phospho-EGF Receptor (Tyr1068) Cat# 3777
Validation	All antibodies were validated by the manufacturer. To ensure the antibodies were specific and sensitive, we further validate our antibodies by testing on multiple cell lines and tissue types with variety of known expression levels.

Eukaryotic cell lines

Policy information about cell lines and Sex and Gender in Research

Cell line source(s)	All glioma sphere models were established at UCLA or UAB, and the information is provided in the methods section and Table S1: Cell line information.<br>Normal Human Astrocytes Lonza Cat# CC-2565<br>HBEC-5i ATCC Cat# CRL-3245<br>157 (Mao et al., 2013)<br>711 (Bhat et al., 2013) See Table S2<br>1051 (Minata et al., 2019) See Table S2<br>1079 (Minata et al., 2019) See Table S2

	TEC15 This study See Table S2<br>TEC14 This study See Table S2<br>293FT Thermofisher Cat#R70007<br>mG7080 (Sadahiro et al., 2018) See Table S4<br>413 (Ghochani et al.,2022) See Table S4<br>408 (Muthukrishnan et al., 2022) N/A<br>PDGFBB derived cells This study N/A
Authentication	Cell lines were extensively validated by whole genome sequencing and cells were monitored regularly by STR analysis.
Mycoplasma contamination	Cell lines were routinely tested for mycoplasma contamination using MycoAlert Mycoplasma detection kit and tested negative.
Commonly misidentified lines (See ICLAC register)	No misidentified cell lines were used in the study.

Animals and other research organisms

Policy information about studies involving animals; ARRIVE guidelines recommended for reporting animal research, and Sex and Gender in Research

Laboratory animals	Strains: C57BL6 mice were used at 8-12 weeks of age.<br>ESM1 knockout mice (Rocha et al., 2014)<br>NOD scid mice -Prkdcscid The Jackson Laboratory Cat# 001303
Wild animals	No wild animals were used in the study.
Reporting on sex	Only female mice were used in the study.
Field-collected samples	N/A
Ethics oversight	All the animal studies were performed according to UCLA ARC (Animal Research Committee) and UAB IACUC (Institutional Animal Care and Use Committee) approved protocol. Mice were housed at AAALAC-approved facilities on a 12-hour light/dark cycle, with food and water available ad libitum.

Note that full information on the approval of the study protocol must also be provided in the manuscript.

Flow Cytometry

Plots

Confirm that:

- [x] The axis labels state the marker and fluorochrome used (e.g. CD4-FITC).
- [x] The axis scales are clearly visible. Include numbers along axes only for bottom left plot of group (a 'group' is an analysis of identical markers).
- [x] All plots are contour plots with outliers or pseudocolor plots.
- [x] A numerical value for number of cells or percentage (with statistics) is provided.

Methodology

Sample preparation	Cells from control and treatment groups were prepared accordingly for Propodium Iodide staining (Cell Cycle analysis) or Caspase 3/7 staining with SYTOX (ThermoFisher).
Instrument	AttuneNxT Flow Cytometer (ThermoFisher Scientific)
Software	FlowJo 10
Cell population abundance	All GBM cells obtained after preparation of single cells were analyzed without separation into different populations.
Gating strategy	The samples were gated by FSC-H and SSC-H to distinguish cells from debris.

- [x] Tick this box to confirm that a figure exemplifying the gating strategy is provided in the Supplementary Information.
