# Supplementary figures and images for: Endothelial-secreted Endocan activates PDGFRA and regulates vascularity and spatial phenotype in glioblastoma
Source: Nat Commun. 2025 Jan 7;16:471. doi: 10.1038/s41467-024-55487-1 (PMC11707362; doi:10.1038/s41467-024-55487-1)

Fig 4a

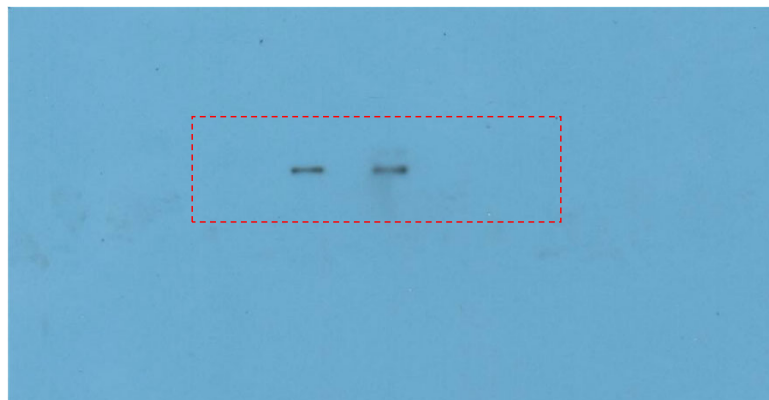

PDGFRA

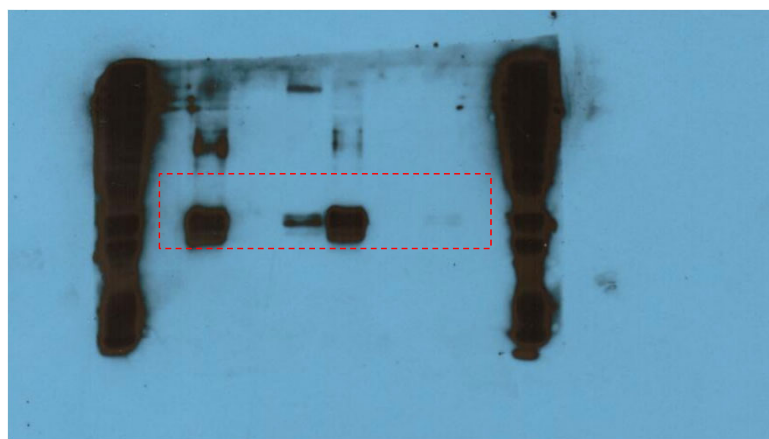

His-tag

Fig 4c

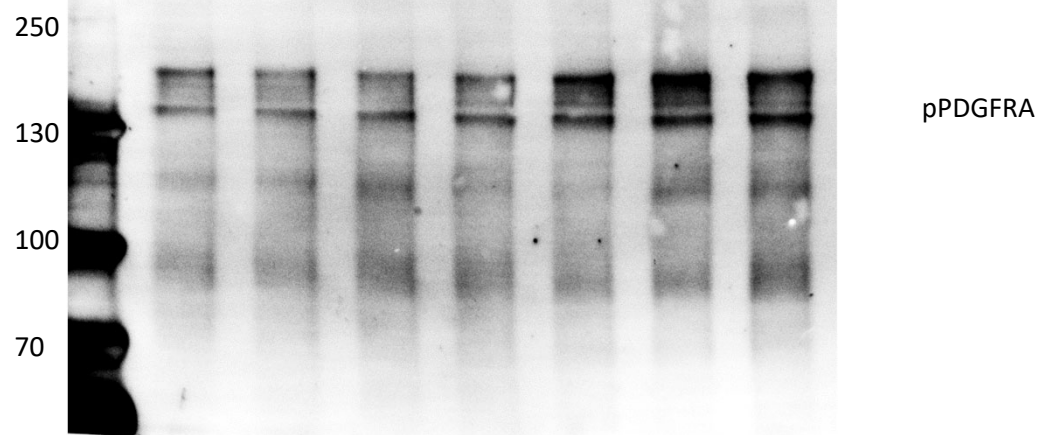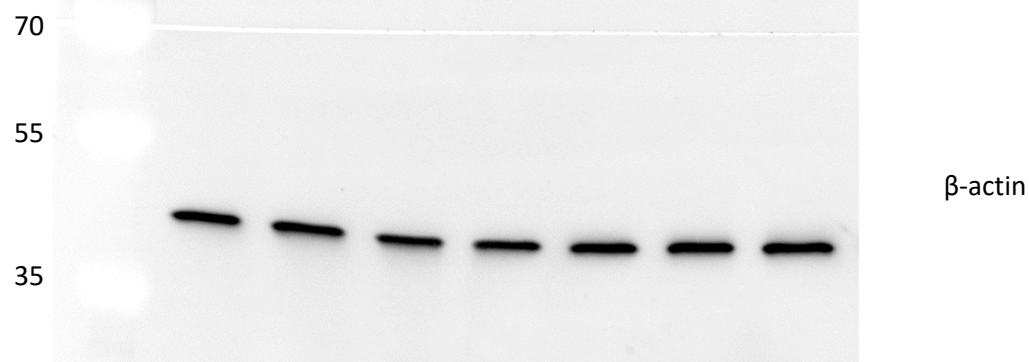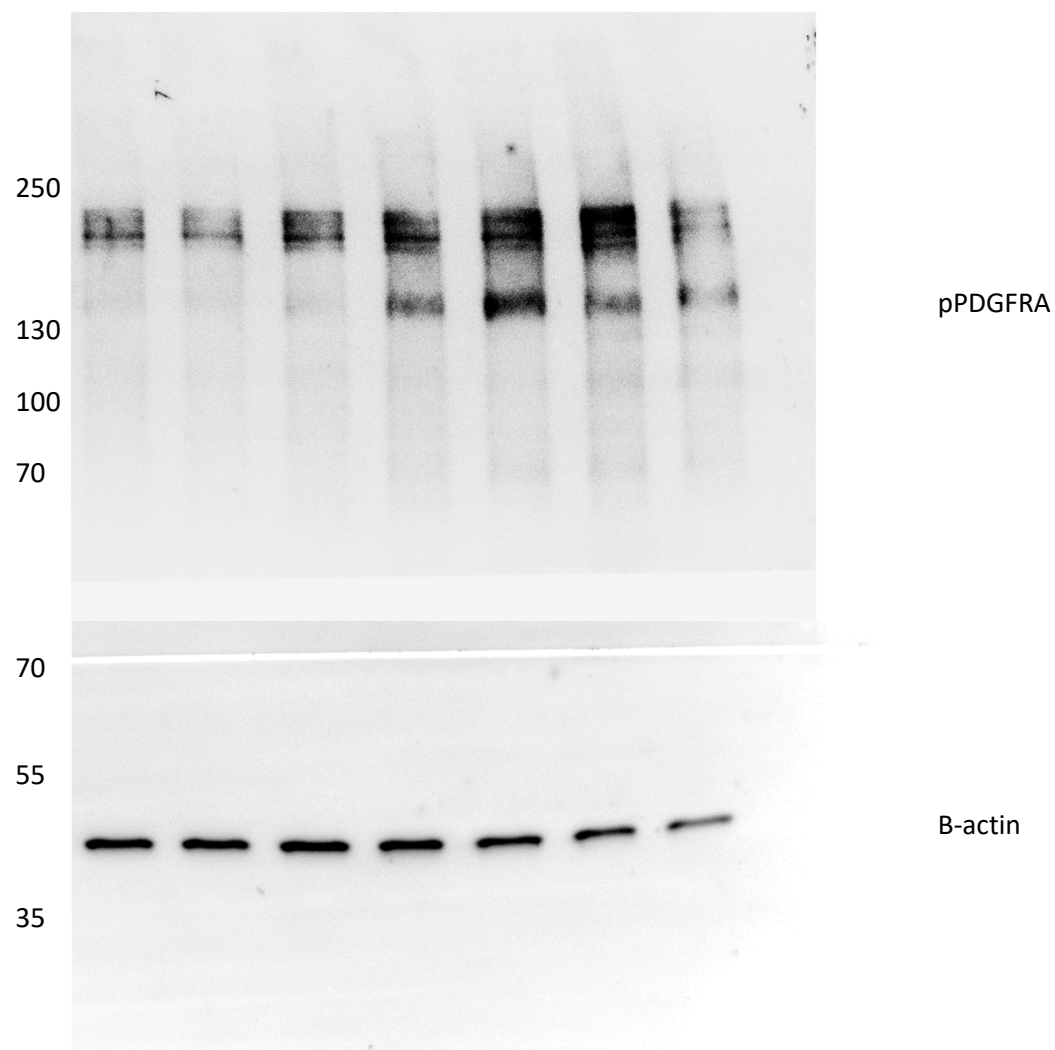

Fig 4d

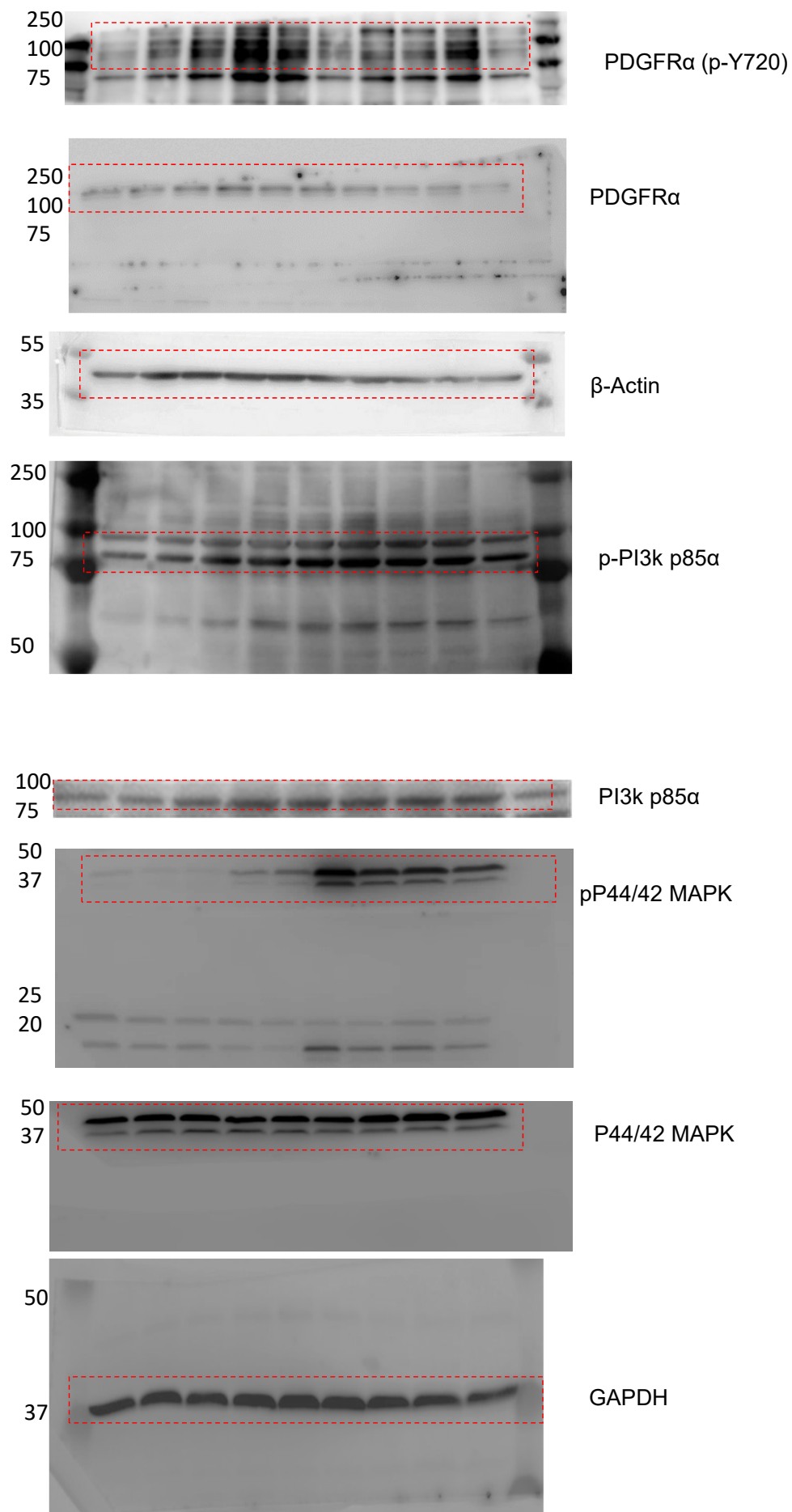

Fig 5e

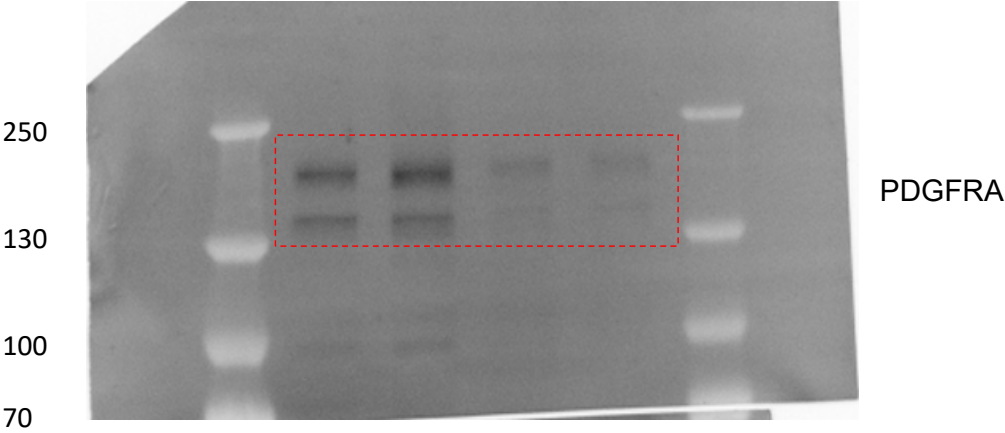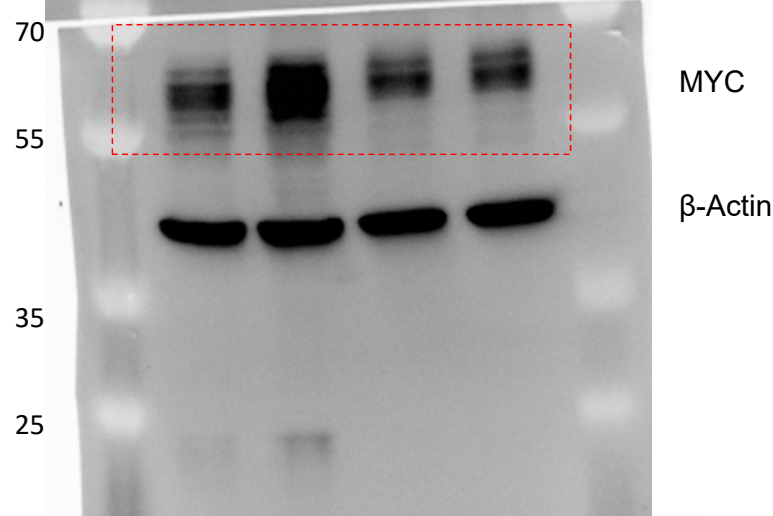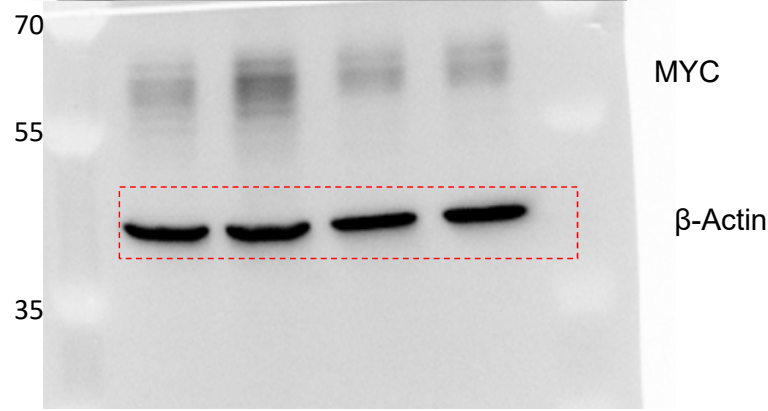

Fig 5g

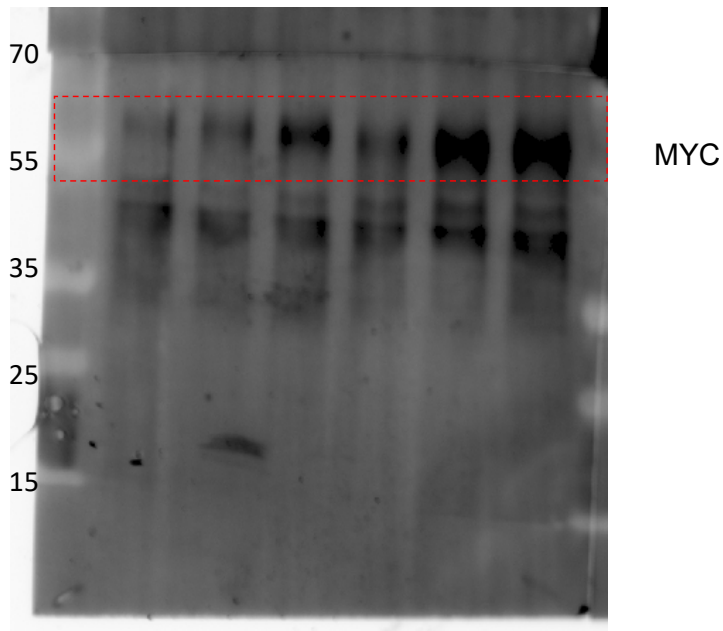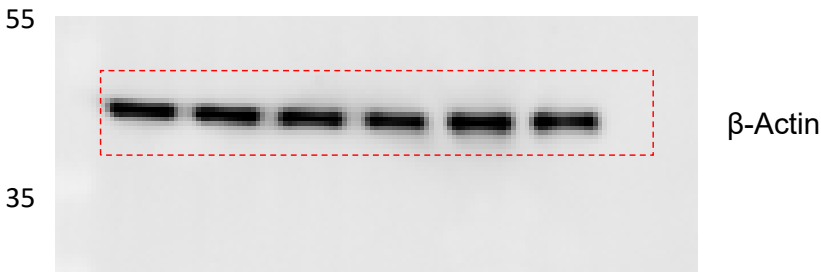

1079 cells

Fig 6a

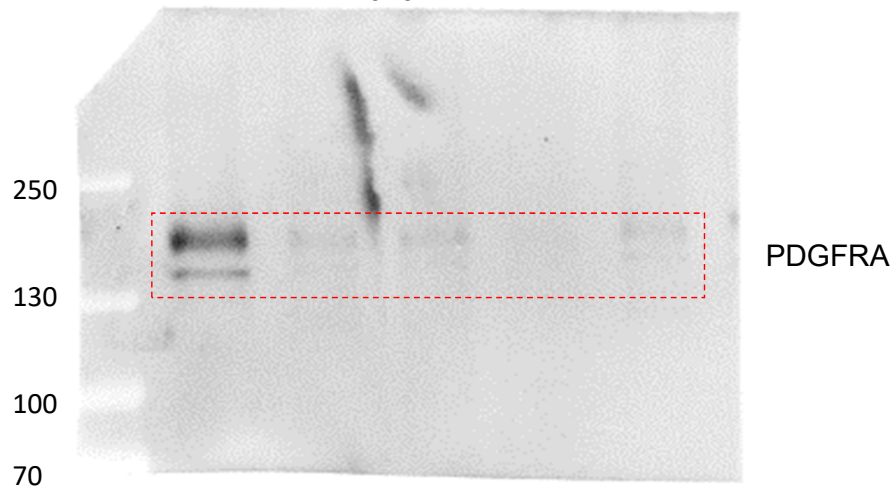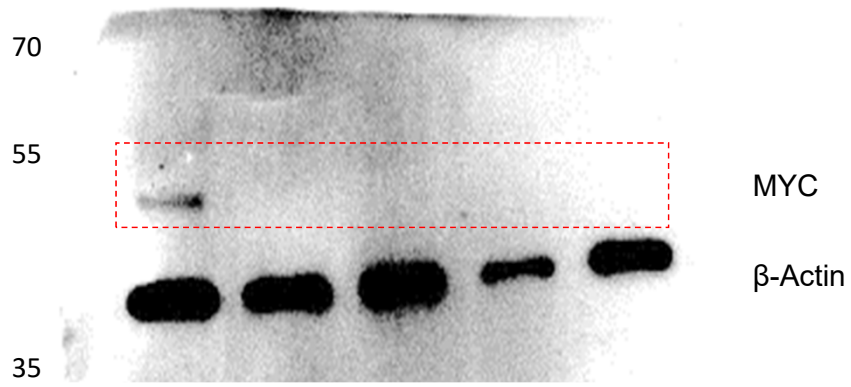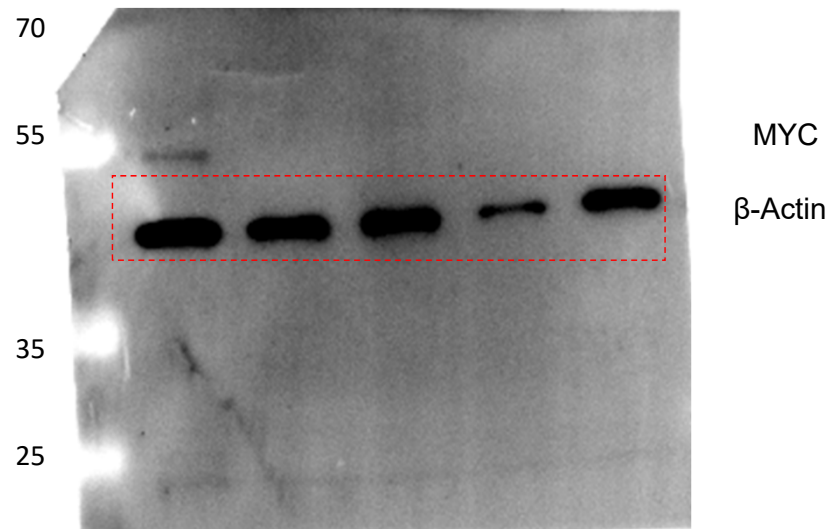

1079 cells

Fig 6c

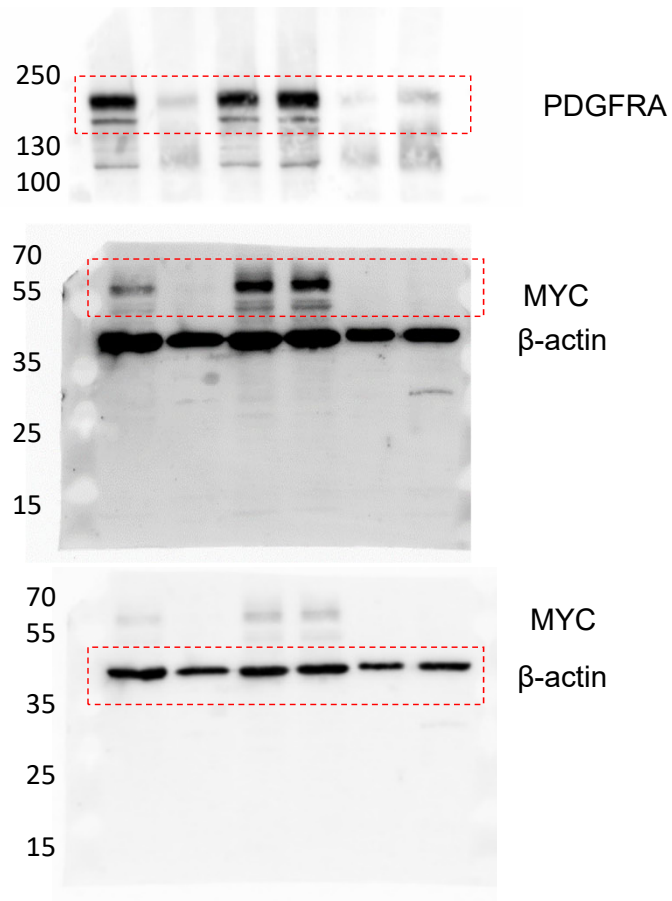

Supplement: Supplementary file 6 — Source Data [file 41467_2024_55487_MOESM6_ESM.zip › Source Data WB.pdf]
